# Self-efficacy in idiopathic inflammatory myopathies: associated factors, profiles and patient-reported outcomes

**DOI:** 10.1093/rheumatology/keag458

**Published:** 2026-08-25

**Authors:** Praggya Yaadav, Lekshmi Minikumari Rahulan, Suhana Hussain, Manali Sarkar, Vincenzo Venerito, Vahed Maroufy, Maria R Pellico, Anne-Marie Russell, Sreoshy Saha, Elena Nikiphorou, Ioannis Parodis, Vidya S Limaye, Dimitri L F da Silva, Samuel K Shinjo, Jessica Day, Aviya L Levy, Karen Cheng, Laura Andreoli, Jasmine Parihar, COVAD Study Group, Odirlei Andre Monticielo, Simone Appenzeller, Binit Vaidya, Raphael Micheroli, Lisa Christ, Oliver Distler, Konstantinos Parperis, A T M Tanveer Hasan, Rada Miskovic, Uzma Rasheed, Babur Salim, Rosalie Magro, Cristina Alexandru, Anca Bobircă, Claudia Cobilinschi, Leandro Gabriel Ferreyra Garrott, Ai Lyn Tan, Nelly Ziade, Tsvetelina Velikova, Abraham Edgar Gracia-Ramos, Masataka Kuwana, Johannes Knitza, Ashima Makol, Carlos Enrique Toro Gutiérrez, Carlo Vinicio Caballero-Uribe, Dzifa Dey, Chris Wincup, Nicola Dalbeth, Gerd-Rüdiger Burmester, Guochun Wang, Lorenzo Cavagna, Vikas Agarwal, Latika Gupta

**Affiliations:** Maharashtra Institute of Medical Sciences and Research, Latur, Maharashtra, India; Department of Clinical Immunology and Rheumatology, Sanjay Gandhi Postgraduate Institute of Medical Sciences, Lucknow, India; Mahatma Gandhi Medical College and Hospital, Jaipur, India; Sir H. N. Reliance Foundation Hospital and Research Centre, Mumbai, Maharashtra, India; University of Bari, Department of Precision and Regenerative Medicine and Ionian Area, Rheumatology Unit, Bari, Italy; Department of Biostatistics and Data Science, School of Public Health, The University of Texas Health Center at Houston School of Public Health, Austin, TX, USA; Department of Clinical Sciences and Community Health, University of Milan, Milan, Italy; Division of Rheumatology, ASST Gaetani Pini-CTO, Milan, Italy; School of Medicine and Health, University of Birmingham, Birmingham, UK; School of Health and Care Professions, University of Exeter, Exeter, UK; ILD Regional Service, University Hospitals Birmingham NHS Trust, Birmingham, UK; Mymensingh Medical College Hospital, Mymensingh, Bangladesh; Centre for Rheumatic Diseases, King’s College London, London, UK; Rheumatology Department, King’s College Hospital, London, UK; Division of Rheumatology, Department of Medicine Solna, Karolinska Institutet, Karolinska University Hospital, and Center for Molecular Medicine (CMM), Stockholm, Sweden; Department of Rheumatology, Faculty of Medicine and Health, Örebro University, Örebro, Sweden; Rheumatology Department, Royal Adelaide Hospital, Adelaide, SA, Australia; Discipline of Medicine, Adelaide University, Adelaide, SA, Australia; Department of Dermatology, University Santo Amaro, Hospital Israelita Albert Einstein, São Paulo, Brazil; Division of Rheumatology, Faculdade de Medicina FMUSP, Universidade de Sao Paulo, Sao Paulo, SP, Brazil; Department of Rheumatology, Royal Melbourne Hospital, Parkville, VIC, Australia; Walter and Eliza Hall Institute of Medical Research, Parkville, VIC, Australia; Department of Medical Biology, University of Melbourne, Parkville, VIC, Australia; Rady Children’s Hospital, University of California San Diego, San Diego, CA, USA; Patient Research Partner/Volunteer, EULAR PARE, Zurich, Switzerland; International Myositis Society, Göttingen, Germany; Patient Advisory Board, Myositis International Health and Research Alliance, New Orleans, Louisiana, USA; Rheumatology and Clinical Immunology Unit, ASST Spedali Civili and University of Brescia, Brescia, Italy; Department of Clinical and Experimental Sciences, University of Brescia, Brescia, Italy; Danish Centre for Expertise in Rheumatology (CeViG), Danish Hospital for Rheumatic Diseases, Sønderborg, Denmark; Department of Regional Health Research, University of Southern Denmark, Odense, Denmark; All India Institute of Medical Science, New Delhi, and National Cancer Institute, Jhajjar, India; Department of Clinical Immunology and Rheumatology, Sanjay Gandhi Postgraduate Institute of Medical Sciences, Lucknow, India; Department of Rheumatology, Royal Wolverhampton Hospitals NHS Trust, Wolverhampton, UK; Department of Inflammation and Ageing, School of Infection, Inflammation and Immunology, University of Birmingham, Edgbaston, UK; Francis Crick Institute, London, UK

**Keywords:** self-efficacy, idiopathic inflammatory myopathies, patient-reported outcomes, self-management, mental health comorbidity, myositis, cluster analysis

## Abstract

**Objectives:**

Self-efficacy (SE) is central to chronic disease self-management but remains poorly characterized in idiopathic inflammatory myopathies (IIMs). We aimed to identify factors associated with SE, characterize SE profiles and examine associations with patient-reported outcomes (PROs) in IIMs.

**Methods:**

Cross-sectional data from 5802 participants across IIMs, other rheumatic diseases (RMDs) and non-rheumatic autoimmune diseases (nRAIDs) were analysed. SE was measured using the Self-Efficacy for Managing Chronic Disease (SEMCD) scale. Hierarchical regression identified factors independently associated with SE, and k-means clustering defined SE health profiles. Associations with PROs were examined in a separate multivariable model.

**Results:**

IIM patients had lower SE (median SEMCD 5.3) than those with RMDs (5.8) and nRAIDs (6.5), with confidence lowest for managing fatigue and physical discomfort. In the fully adjusted integrated model (R² = 0.222), mental health comorbidity (B = −0.898) and financial difficulty (B = −0.734) were most strongly associated with lower SE, while regular exercise (B = 0.770) and family support (B = 0.150) were associated with higher SE. The PRO model explained 48.3% of SE variance (R² = 0.483); resilience was the strongest positive association, while fatigue and pain were negatively associated. Four SE health profiles were identified, including a clinically distinctive resilient-despite-burden subgroup (24%) with high symptom burden but preserved SE and mental health. Higher SE was associated with better physical and mental health, greater resilience and life satisfaction, and lower fatigue, pain and loneliness (all *P *< 0.001).

**Conclusion:**

SE in IIMs is associated with modifiable socioeconomic and psychological factors and with better health outcomes. Profile-based stratification may help identify vulnerable subgroups and guide targeted interventions.

## Graphical abstract

**Graphical Abstract:**
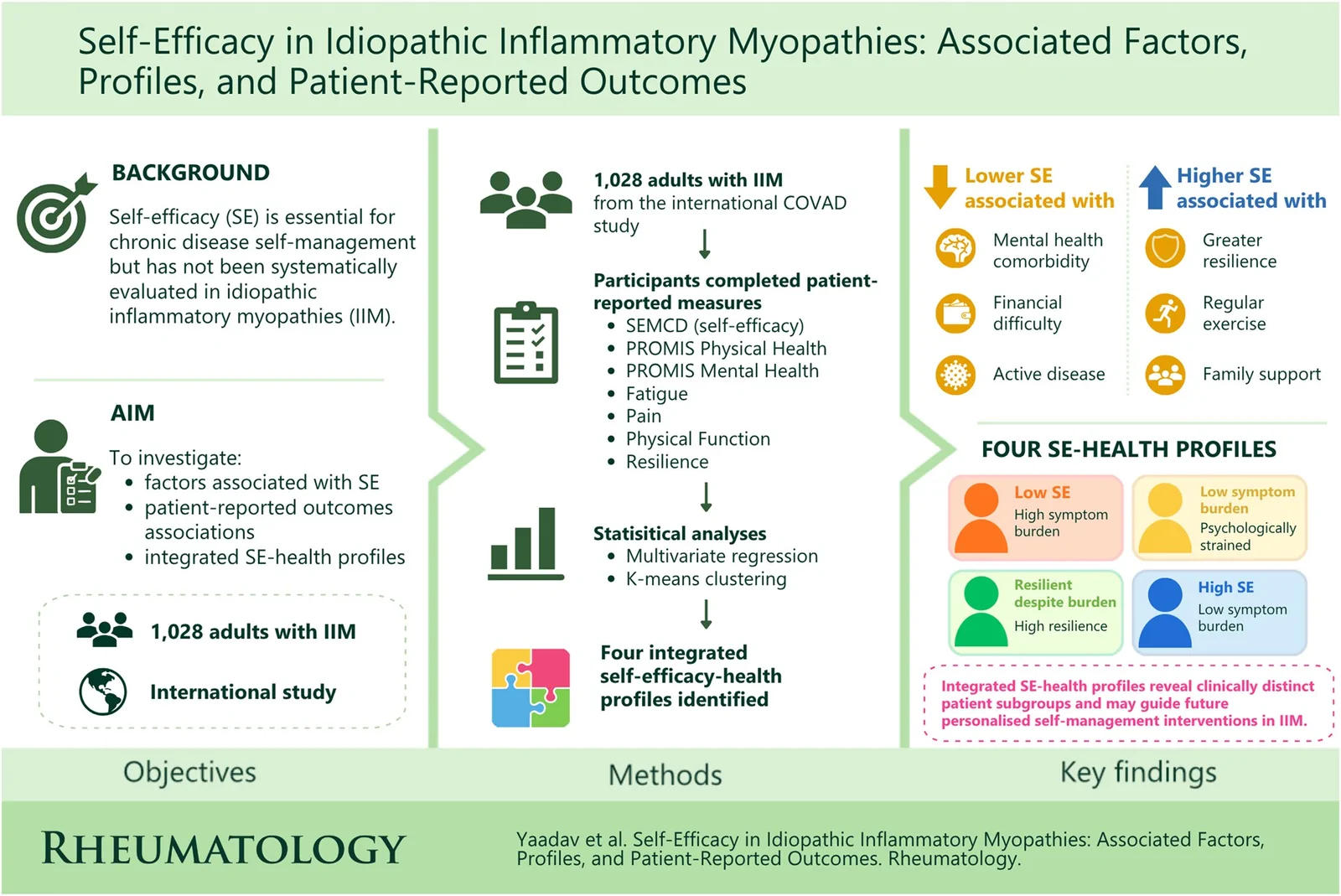

*Rheumatology* key messages

- Self-efficacy (SE) in idiopathic inflammatory myopathies (IIMs) is lower than in other autoimmune diseases and is shaped by modifiable factors.
- Four integrated SE health profiles were identified, including a resilient-despite-burden subgroup with preserved SE.
- Profile-based stratification may help identify vulnerable subgroups for future, prospectively evaluated self-management support.

## Introduction

Idiopathic inflammatory myopathies (IIMs) are heterogeneous systemic autoimmune conditions characterized by chronic muscle weakness, inflammatory muscle infiltrates, and multi-organ involvement affecting the skin, joints, lungs, heart and gastrointestinal tract [1]. Although the reported incidence of IIMs has increased in the 21st century, whether this reflects a true epidemiological change remains uncertain [2]. High comorbidity burden [3], persistent physical and psychological symptoms, and the need for multiple medications impose substantial treatment burden [4]. Despite immunosuppressive therapy, up to 80% of patients continue to experience disease activity and functional impairment [5], with fatigue, pain and reduced function often persisting despite apparently controlled disease [6]. Effective self-management is therefore essential to optimize quality of life (QoL) [7].

Self-efficacy (SE), defined as an individual’s confidence in their capacity to successfully perform an activity [8], is an important psychological mechanism underlying effective self-management in chronic conditions [9–11]. Higher SE has been associated with less pain, better physical function and improved self-management in SSc and RA [12–14]. However, factors associated with SE and its relationship with patient-reported outcomes (PROs) remain poorly understood in IIMs despite their uniquely demanding self-management requirements [9, 15, 16].

This study examined factors associated with SE, characterized SE profiles, and assessed associations between SE and PROs in adults with IIMs using a large international dataset. We hypothesized that in IIMs SE would be lower than in other autoimmune diseases, independently associated with modifiable factors including mental health comorbidity and financial difficulty, and that profile-based stratification would identify clinically meaningful subgroups.

## Methods

### Study design and data source

Data were drawn from the Collating the Voice of People with Autoimmune Diseases dataset, an international, cross-sectional e-survey conducted between February 2024 and October 2025. The instrument was developed by a multidisciplinary steering committee, including rheumatologists, researchers, social scientists, psychologists and patient research partners. It was translated into multiple languages using validated forward–backward translations and administered via SurveyMonkey; the full protocol has been published previously [17]. Informed consent was obtained electronically, all responses were anonymous and ethical approval was obtained from the Institutional Ethics Committee of SGPGIMS, Lucknow, India (226014), with additional local approvals obtained as required [18]. The Checklist for Reporting Results of the Internet E-Surveys (CHERRIES) was followed [19].

### Data extraction and study population

Data were extracted following survey closure in October 2025. Participants were eligible if aged over 18 years. Data were cleaned, and responses were excluded if key demographic variables (age or sex) were missing, diagnosis was uncertain, self-diagnosed or not confirmed by a specialist, Self-Efficacy for Managing Chronic Disease (SEMCD) scale data did not meet the minimum completion threshold or if participants had cognitive impairment. Disease diagnoses were based on participant self-report of a physician-assigned diagnosis. Participants reporting self-diagnosis or diagnosis by a non-specialist were excluded. Self-reported diagnoses were cross-checked against disease manifestations and immunosuppressive medication use to minimize misclassification. As this was a multinational e-survey, independent verification using formal classification criteria, antibody profiles or medical records was not available. The data-cleaning protocol is provided in the Supplementary Material.

Participants were categorized into three groups: (i) IIMs, including DM, PM, IBM, necrotizing autoimmune myopathy (NAM), anti-synthetase syndrome (ASyS) and overlap myositis (OM); (ii) other rheumatic diseases (RMDs), including RA, SLE, Sjögren’s disease, SSc and related conditions; and (iii) non-rheumatic autoimmune diseases (nRAIDs), including type 1 diabetes mellitus, Hashimoto’s thyroiditis, psoriasis and related conditions. A total of 57 IIM patients could not be assigned to a specific subtype and were included in overall IIM analyses but excluded from subtype-level comparisons. Countries were categorized by the Human Development Index (HDI) as very high, high, medium or low [20]. Three levels of multimorbidity were defined. Basic multimorbidity denoted the presence of two or more non-rheumatic chronic conditions. Complex multimorbidity required the co-occurrence of three or more chronic conditions spanning at least three distinct physiological systems. Autoimmune multimorbidity was defined as three or more distinct systemic autoimmune diseases. Active disease was defined as a Patient Global Disease Activity score >1 combined with at least one of the following: evidence of inflammation on imaging or laboratory tests; CS use >10 mg/day (prednisone equivalent); or i.v./intralesional steroids in the past 3 months [17].

### Self-efficacy measurement

SE was assessed using the SEMCD scale, a validated 6-item instrument measuring confidence in managing fatigue, pain, physical discomfort, mental distress, healthcare utilization and daily functioning. Items are rated from 1 (‘not at all confident’) to 10 (‘totally confident’), with higher scores indicating greater SE [21]. The SEMCD has been validated in chronic conditions, including SSc [22, 23] and RA [24]. The overall score is the mean of six items, provided at least four are completed [21]; participants with non-consecutive item selections (i.e. skipping items mid-scale then resuming, which may indicate inattentive responding) were excluded.

Two complementary approaches were used to classify SE: (i) percentile-based thresholds to define relatively low and high SE groups, and (ii) data-driven k-means clustering to identify naturally occurring SE health profiles. Participants were classified as high (>67th percentile; SEMCD >6.3) or low (<33rd percentile; SEMCD ≤4.5) SE based on the distribution of SEMCD scores within the IIM patient group. Internal consistency was assessed using Cronbach’s alpha, with ≥0.70 considered acceptable and ≥0.90 excellent [25].

### Patient-reported outcomes

PROs included overall physical health [Patient-Reported Outcomes Measurement Information System (PROMIS) GPH], physical function (PROMIS PF4a), mental health (PROMIS GMH), life satisfaction [Satisfaction With Life Scale (SWLS)], fatigue [visual analogue scale (VAS)], pain (VAS), loneliness (UCLA Loneliness Scale) and resilience [Brief Resilience Scale (BRS)]. Additional clinical variables included disease activity, disease duration, comorbidities, and medication use. Demographic and socioeconomic variables included age, sex, ethnicity, education, household income, employment, household size, marital status, family support [Family Adaptation, Partnership, Growth, Affection, and Resolve (APGAR)] and HDI category.

### Statistical analysis

All statistical analyses were conducted using R [26] via RStudio (Version 4.5) and Jamovi (Version 2.7) [27]. Continuous variables were reported as median and interquartile range (IQR); categorical variables as frequencies and percentages (*n*, %). Group comparisons used the χ^2^ test, Fisher’s exact test, Student’s *t*-test or Mann–Whitney *U* test as appropriate; the Kruskal–Wallis test was used for comparisons across multiple groups. Spearman’s correlation coefficient was used to assess associations between variables. Differences in SE across disease groups (IIM, other RMD and nRAID) were assessed using analysis of covariance, adjusting for age, disease duration, comorbidity burden (Functional Comorbidity Index) and sex, with IIM as the reference category. A sensitivity analysis additionally adjusted for ethnicity and country-level HDI.

**Figure 1:**
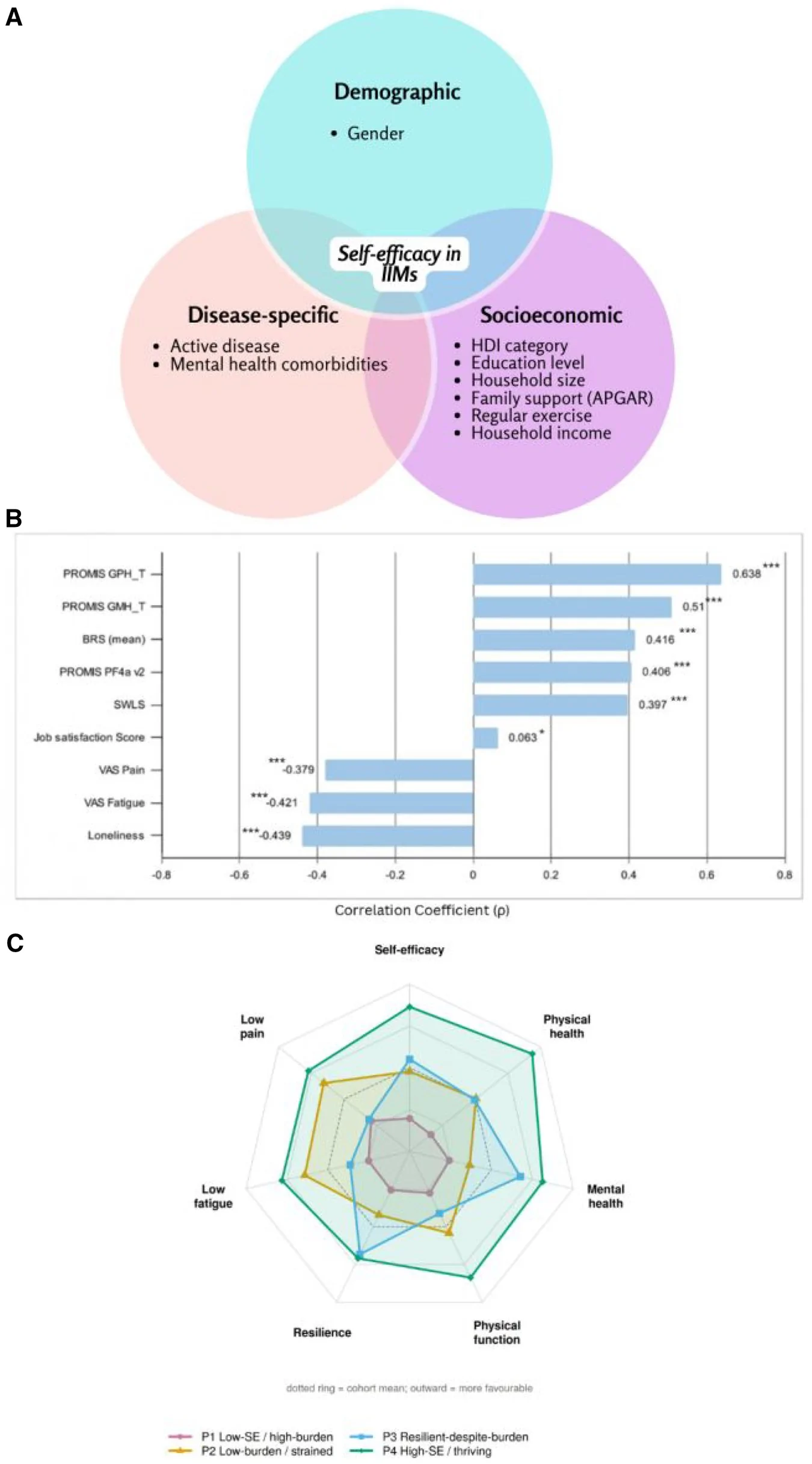
(**A**) Factors independently associated with SE in IIMs identified in the fully adjusted integrated model; (**B**) correlation of PROs with SE in IIMs (*P < 0.05; **P < 0.01; ***P < 0.001); (**C**) clinical characteristics across K-means derived SE health profiles in IIMs (Radar plot comparing the four integrated SE health profiles [P1, low-SE/high-burden (pink); P2, low-burden/strained (orange); P3, resilient-despite-burden (blue); P4, high-SE/thriving (green)] across SE, physical health, mental health, physical function, resilience, low fatigue and low pain, standardized relative to the cohort mean (dotted ring). P4 shows the largest area across nearly all domains; P3 shows a distinctive pattern of preserved SE and mental health despite high fatigue and pain Figure generated using RStudio. IIM: idiopathic inflammatory myopathies; HDI: Human Development Index; APGAR: Adaptation, Partnership, Growth, Affection, and Resolve; PRO: patient-reported outcomes; PROMIS: Patient-Reported Outcome Measurement Information System; GMH_T: Global Mental Health T-score; GPH_T: Global Physical Health T-score; PF4a: Physical Function Form 4a; VAS: visual analogue scale; BRS: Brief Resilience Scale; SWLS: Satisfaction With Life Score; ρ: Spearman’s Rho; SE: self-efficacy; SEMCD: Self-Efficacy for Managing Chronic Disease

Variables for inclusion in the regression models were selected *a priori* based on clinical relevance and prior literature. To preserve interpretability and group conceptually related factors, hierarchical multivariable linear regression was performed using three pre-specified domain models: (i) demographic, (ii) disease-specific and (iii) socioeconomic (Supplementary Table S4A). Factors retained in these domain models were combined into a fully adjusted integrated model, which constituted the primary analysis (Supplementary Table S4B; Fig. 1A). To verify that estimates were not dependent on this selection step, a sensitivity analysis was performed in which all *a priori* variables were entered simultaneously (forced entry). Multicollinearity was assessed using variance inflation factors (VIF). Education was grouped as below graduate (primary/secondary), undergraduate and postgraduate for regression analysis. A separate multivariable linear regression model examined associations between PROs and SE, including fatigue (VAS), pain (VAS), physical function (PROMIS PF4a), mental health (PROMIS GMH) and resilience (BRS) as independent variables (Supplementary Table S4C). Results are reported as unstandardized beta coefficients (B) with 95% CI. Model fit was assessed using R².

K-means clustering was applied to z-standardized SEMCD scores together with six PROs (PROMIS physical and mental health, fatigue VAS, pain VAS, resilience and physical function). The number of clusters was determined by the elbow and silhouette coefficient alongside clinical interpretability and corroborated by Ward hierarchical clustering. Profiles were validated against external variables not entered into the clustering (life satisfaction, loneliness, mental health comorbidity, income adequacy, disease activity and IIM subtype).

Analyses were restricted to the 1028 IIM participants meeting inclusion criteria. Missing data were not imputed; each model used complete-case (listwise) analysis, and denominators for descriptive statistics reflect available responses. Within the IIM cohort, missingness on the variables entered into the regression and cluster analyses was minimal. All demographic, socioeconomic, lifestyle and PRO variables, including the SEMCD outcome, were complete (0% missing), with the exception of HDI category (8/1028; 0.8%) and IIM subtype [38/1028 unrecorded (3.7%); a further 19 JDM cases were excluded from adult subtype-level analyses, so 57 (5.5%) were not included in subtype-stratified models]. Consequently, the effective sample sizes were 1028 for the demographic and PRO models and the k-means cluster analysis, 971 for the disease-specific model, 1020 for the socioeconomic model and 964 for the fully adjusted integrated model (Supplementary Table S5).

## Results

### Baseline characteristics

The study included a total of 5802 participants: 1028 with IIMs, 4250 with RMDs and 524 with nRAIDs (Supplementary Fig. S1). IIM patients were older (median 60.0 years, IQR 47.8–70.0) than those with RMDs (50.0 years, IQR 40.0–61.0) and nRAIDs (40.0 years, IQR 32.0–52.0). Female predominance was observed across all groups (IIM 72.7%, RMDs 78.1%, nRAIDs 85.5%). Most IIM patients were Caucasian/White (73.4%), resided in very high HDI countries (74.8%) and most commonly reported secondary school education (44.0%). Disease duration was shorter in IIMs (median 7.0 years, IQR 3.0–16.0) than in RMDs (median 11.0 years, IQR 4.0–20.0) and nRAIDs (median 9.0 years, IQR 4.0–18.0), and a lower proportion had inactive disease compared with RMDs (52.5% *vs* 59.7%). IIM patients demonstrated a significantly greater prevalence of complex multimorbidity (15.0%) and autoimmune multimorbidity (14.7%), and higher rates of mental health comorbidities (33.6%) than RMDs, though comparable to nRAIDs (Table 1).

**Table 1:** Characteristics of the overall respondents.

Characteristic	IIM (*n* = 1028)	RMD (*n* = 4250)	nRAID (*n* = 524)	*P* (IIM *vs* RMD)	*P* (IIM *vs* nRAID)
(A) Demographic factors					
Age (years), median (IQR)	60.0 (47.8–70.0)	50.0 (40.0–61.0)	40.0 (32.0–52.0)	<0.001	<0.001
Female, *n* (%)	747 (72.7)	3320 (78.1)	448 (85.5)	<0.001	<0.001
Ethnicity, *n* (%)					
Caucasian/White	755 (73.4)	2073 (48.8)	323 (61.6)	<0.001	<0.001
Asian	147 (14.3)	933 (22.0)	47 (9.0)	<0.001	0.003
Hispanic	14 (1.4)	310 (7.3)	45 (8.6)	<0.001	<0.001
Others	112 (10.9)	934 (22.0)	109 (20.8)	<0.001	<0.001
Level of education, *n* (%)*					
Secondary school	452 (44.0)	1462 (34.4)	97 (18.5)	<0.001	<0.001
Undergraduate degree	344 (33.5)	1546 (36.4)	182 (34.7)	0.076	0.605
Primary school	80 (7.8)	228 (5.4)	60 (11.5)	0.002	0.008
Postgraduate degree	63 (6.1)	609 (14.3)	154 (29.4)	<0.001	<0.001
Self-efficacy					
SEMCD, median (IQR)	5.3 (4.0–7.0)	5.8 (4.5–7.3)	6.5 (4.8–8.3)	<0.001	<0.001
(B) Disease-specific factors					
Active disease, *n* (%)	488 (47.5)	1712 (40.3)	113 (21.6)	<0.001	<0.001
Disease activity VAS	5.0 (4.0–7.0)	5.0 (3.0–6.0)	4.0 (2.0–6.0)	<0.001	<0.001
Disease damage VAS	6.0 (4.0–8.0)	5.0 (3.0–7.0)	4.0 (2.0–6.0)	<0.001	<0.001
Disease duration (years)	7.0 (3.0–16.0)	11.0 (4.0–20.0)	9.0 (4.0–18.0)	<0.001	0.002
Medications, *n* (%)					
Glucocorticoids >10 mg/day	124 (12.1)	326 (7.7)	53 (10.1)	<0.001	0.290
csDMARDs	527 (51.3)	1786 (42.0)	80 (15.3)	<0.001	<0.001
bDMARDs	259 (25.2)	944 (22.2)	53 (10.1)	0.045	<0.001
tsDMARDs	33 (3.2)	254 (6.0)	5 (1.0)	0.001	0.011
Comorbidities, *n* (%)					
Complex multimorbidity	154 (15.0)	400 (9.4)	35 (6.7)	<0.001	<0.001
Autoimmune multimorbidity	151 (14.7)	161 (3.8)	0 (0.0)	<0.001	<0.001
Mental health comorbidity	345 (33.6)	1259 (29.6)	195 (37.2)	0.015	0.170
Cancer	189 (18.4)	326 (7.7)	46 (8.8)	<0.001	<0.001
Functional comorbidity index	1.0 (0.0–3.0)	1.0 (0.0–2.0)	1.0 (0.0–2.0)	0.003	<0.001
(C) Socioeconomic factors					
Country of residence, *n* (%)					
Very high HDI	769 (74.8)	1980 (46.6)	318 (60.7)	<0.001	<0.001
High HDI	110 (10.7)	1062 (25.0)	148 (28.2)	<0.001	<0.001
Medium HDI	134 (13.0)	954 (22.4)	50 (9.5)	<0.001	0.054
Low HDI	15 (1.5)	254 (6.0)	8 (1.5)	<0.001	0.865
Cohabitation status, *n* (%)*					
Married/living as married	718 (69.8)	2909 (68.4)	347 (66.2)	0.407	0.162
Single	116 (11.3)	707 (16.6)	121 (23.1)	<0.001	<0.001
Divorced	81 (7.9)	284 (6.7)	30 (5.7)	0.197	0.146
Widow	64 (6.2)	152 (3.6)	6 (1.1)	<0.001	<0.001
Separated	34 (3.3)	111 (2.6)	11 (2.1)	0.264	0.237
Household income, *n* (%)					
Living comfortably	520 (50.6)	1975 (46.5)	219 (41.8)	0.020	0.001
Getting by	331 (32.2)	1454 (34.2)	220 (42.0)	0.235	<0.001
Difficult	116 (11.3)	575 (13.5)	66 (12.6)	0.062	0.499
Very difficult	61 (5.9)	246 (5.8)	19 (3.6)	0.917	0.068
Total members in household	2.0 (2.0–3.0)	3.0 (2.0–4.0)	3.0 (2.0–4.0)	<0.001	<0.001

*P*-values were assessed by χ² test for categorical variables and by Student’s *t*-test or Mann–Whitney *U* test for normally and non-normally distributed continuous variables, respectively. *P* < 0.05 statistically significant. *Percentages are based on available data and may not sum to 100% due to missing responses and rounding. IIM: idiopathic inflammatory myopathies; RMD: other rheumatic disease; IQR: interquartile range; HDI: Human Development Index; csDMARDs: conventional synthetic DMARDs; bDMARDs: biologic DMARDs; tsDMARDs: targeted synthetic DMARDs; VAS: visual analogue scale; SEMCD: Self-Efficacy for Managing Chronic Disease.

Among IIM subtypes, IBM patients were the oldest with the longest disease duration (median 10.0 years, IQR 7.0–14.0), while NAM had the shortest (median 4.0 years, IQR 2.0–7.0). Active disease was lowest in IBM (37.8%) and highest in PM (58.2%) and NAM (56.9%). Complex multimorbidity was most frequent in ASyS (22.1%), autoimmune multimorbidity most prevalent in OM (45.2%) followed by ASyS (30.0%), and mental health comorbidities were highest in OM (45.2%), NAM (39.7%) and DM (39.2%). IBM demonstrated the lowest comorbidity burden overall (Table 2).

**Table 2:** Characteristics of the respondents across IIM subtypes.

Variable	DM (*n* = 265)	ASyS (*n* = 140)	PM (*n* = 141)	IBM (*n* = 283)	NAM (*n* = 58)	OM (*n* = 84)	*P*-value
(A) Self-efficacy							
SEMCD, median (IQR)	5.5 (4.2–6.8)	5.2 (4.2–6.3)	5.3 (3.8–6.8)	5.3 (4.0–7.0)	5.9 (3.7–8.1)	4.7 (3.2–6.2)	0.088
High self-efficacy (>6.33), *n* (%)	80 (30.2)	33 (23.6)	50 (35.5)	97 (34.3)	22 (37.9)	18 (21.4)	0.045
(B) Disease-specific factors							
Age (years), median (IQR)	45.0 (34.0–57.0)	41.0 (29.8–53.0)	49.0 (37.0–57.0)	61.0 (52.0–68.0)	53.0 (43.0–60.0)	32.5 (25.0–45.0)	<0.001
Disease duration (years), median (IQR)	6.0 (3.0–10.0)	5.0 (3.0–9.0)	6.5 (4.0–11.0)	10.0 (7.0–14.0)	4.0 (2.0–7.0)	5.0 (3.0–9.0)	<0.001
Active disease, *n* (%)	125 (47.2)	76 (54.3)	82 (58.2)	107 (37.8)	33 (56.9)	39 (46.4)	<0.001
Medication, *n* (%)							
Steroids >10 mg/day	42 (15.8)	25 (17.9)	32 (22.7)	12 (4.2)	11 (19.0)	7 (8.3)	<0.001
csDMARDs	192 (72.5)	65 (46.4)	56 (39.7)	49 (17.3)	42 (72.4)	43 (51.2)	<0.001
tsDMARDs	20 (7.5)	7 (5.0)	2 (1.4)	0 (0.0)	0 (0.0)	3 (3.6)	<0.001
bDMARDs	86 (32.5)	41 (29.3)	15 (10.6)	30 (10.6)	29 (50.0)	13 (15.5)	<0.001
Comorbidities, *n* (%)							
Basic multimorbidity	135 (50.9)	79 (56.4)	72 (51.1)	145 (51.2)	25 (43.1)	44 (52.4)	<0.001
Complex multimorbidity	49 (18.5)	31 (22.1)	15 (10.6)	28 (9.9)	10 (17.2)	13 (15.5)	0.027
Autoimmune multimorbidity	42 (15.8)	42 (30.0)	27 (19.1)	37 (13.1)	7 (12.1)	38 (45.2)	<0.001
Mental health comorbidities	104 (39.2)	41 (29.3)	41 (29.1)	72 (25.4)	23 (39.7)	38 (45.2)	<0.001
Functional Comorbidity Index, median (IQR)	1.0 (0.0–3.0)	1.0 (0.0–2.0)	1.0 (0.0–3.0)	1.0 (0.0–2.0)	2.0 (1.0–3.0)	1.0 (0.0–2.8)	<0.001
(C) Socioeconomic factors							
Country of residence, *n* (%)							
Very high HDI	234 (88.3)	74 (52.9)	131 (92.9)	281 (99.3)	50 (86.2)	44 (52.4)	<0.001
High HDI	17 (6.4)	7 (5.0)	4 (2.8)	2 (0.7)	7 (12.1)	24 (28.6)	
Medium HDI	12 (4.5)	59 (42.1)	4 (2.8)	0 (0.0)	0 (0.0)	11 (13.1)	
Low HDI	2 (0.8)	0 (0.0)	1 (0.7)	0 (0.0)	0 (0.0)	0 (0.0)	
Cohabitation status, *n* (%)*							
Married or living as married	182 (68.7)	103 (73.6)	107 (75.9)	211 (74.6)	38 (65.5)	42 (50.0)	<0.001
Single	41 (15.5)	16 (11.4)	12 (8.5)	21 (7.4)	5 (8.6)	25 (29.8)	
Divorced	23 (8.7)	5 (3.6)	5 (3.5)	22 (7.8)	8 (13.8)	3 (3.6)	
Widow	13 (4.9)	7 (5.0)	9 (6.4)	25 (8.8)	2 (3.4)	5 (6.0)	
Separated	5 (1.9)	9 (6.4)	8 (5.7)	4 (1.4)	5 (8.6)	6 (7.1)	
People in household, median (IQR)	3.0 (2.0–4.0)	3.0 (2.0–4.0)	3.0 (2.0–4.0)	2.0 (2.0–3.0)	3.0 (2.0–4.0)	3.0 (2.0–5.0)	<0.001

*P*-values were assessed by χ² test for categorical variables and by analysis of variance or Kruskal–Wallis test for normally and non-normally distributed continuous variables, respectively. *P* < 0.05 statistically significant. The sum of subtype totals (*n* = 971) is less than the total IIM sample (*n* = 1028) as 57 patients could not be classified into a specific IIM subtype and were therefore excluded from subtype-level analyses. *Percentages are based on available data and may not sum to 100% due to missing responses and rounding. IIM: idiopathic inflammatory myopathies; NAM: necrotizing autoimmune myopathy; ASyS: anti-synthetase syndrome; OM: overlap myositis; IQR: interquartile range; HDI: Human Development Index; SEMCD: Self-Efficacy for Managing Chronic Disease; csDMARDs: conventional synthetic DMARDs; bDMARDs: biologic DMARDs; tsDMARDs: targeted synthetic DMARDs.

### Self-efficacy across groups and domains

The SEMCD scale demonstrated excellent internal consistency in IIM patients (Cronbach’s alpha 0.917, 95% CI 0.909–0.925). In unadjusted comparisons, IIM patients had significantly lower SE (median SEMCD 5.3) than those with RMDs (5.8) and nRAIDs (6.5). After adjustment for age, disease duration, comorbidity burden and sex, SE differed significantly across disease groups (F = 50.1, *P *< 0.001). IIM patients had the lowest adjusted SE (adjusted mean 5.46), compared with other RMD (5.92; adjusted β = +0.47, 95% CI 0.32–0.61) and nRAID (6.57; adjusted β = +1.11, 95% CI 0.89–1.33); the difference between the latter two groups was also significant (β = −0.65, 95% CI −0.83 to −0.46). These differences were robust in a sensitivity model additionally adjusting for ethnicity and HDI. Using percentile-based thresholds, 323 IIM patients had low SE (≤4.5; 33rd percentile) and 355 had high SE (>6.3; 67th percentile). SE was not uniform across all management domains: confidence was highest for managing emotional distress, reducing healthcare visits and managing the condition beyond medication, and lowest for managing fatigue, other symptoms and physical discomfort (Supplementary Table S1).

SE was significantly associated with multiple PROs in IIM patients. Strong positive correlations were observed with physical health (PROMIS GPH, ρ = 0.638), mental health (PROMIS GMH, ρ = 0.51), physical function (PROMIS PF-4a v2, ρ = 0.406), life satisfaction (SWLS, ρ = 0.397) and resilience (BRS, ρ = 0.416; all *P* < 0.001). Inverse associations were observed with loneliness (ρ = −0.439), fatigue (VAS, ρ = −0.421) and pain (VAS, ρ = −0.379; all *P* < 0.001) (Fig. 1B, Supplementary Table S2).

### Factors associated with self-efficacy in IIMs

Subgroup comparisons revealed substantial variations in SE across socioeconomic, demographic and clinical factors (Supplementary Table S3). SE declined progressively with increasing financial difficulty (*P* < 0.001), with median SEMCD ranging from 5.83 (IQR 4.50–7.50) in those living comfortably to 4.17 (IQR 3.33–5.17) in those finding it very difficult. Active disease was associated with lower SE (5.17, IQR 3.83–6.67) compared with inactive disease (5.67, IQR 4.17–7.33; *P* = 0.001). Patients receiving high-dose glucocorticoids (>10 mg/day) had lower SE scores (5.0, IQR 3.50–6.83) than those on lower doses (5.33, IQR 4.00–7.00); however, this difference did not reach statistical significance (*P* = 0.053). Education level demonstrated a non-linear association with SE (*P* = 0.018). While postgraduate education was associated with the highest SE (5.83, IQR 4.17–7.33), relatively high SE was also observed among participants with no formal schooling (5.75, IQR 4.08–7.50). In adjusted analysis, education below graduate level was independently associated with lower SE (B = −0.395; *P* = 0.002). Gender was not associated with SE in unadjusted analyses (*P* = 0.226); however, in the fully adjusted model, female sex emerged as a significant factor positively associated with SE (B = 0.472, *P* = 0.002).

#### Demographic and disease-related factors

In the fully adjusted integrated model (R² = 0.222, *P* < 0.001), mental health comorbidities showed the strongest disease-related negative association with SE (B = −0.898, 95% CI −1.156 to −0.640; *P* < 0.001). Active disease remained independently associated with lower SE (B = −0.307, 95% CI −0.544 to −0.070; *P* = 0.011). Among IIM subtypes, OM showed a trend toward lower SE compared with DM (B = −0.452; *P* = 0.064), although this did not reach statistical significance. Age, ethnicity, steroid use and disease duration were not significantly associated with SE (Supplementary Table S4A).

#### Socioeconomic and lifestyle factors

Financial difficulty showed the strongest overall negative association with SE (B = −0.734, 95% CI −1.062 to −0.405; *P* < 0.001). Lower education was independently associated with reduced SE in patients below graduate level (B = −0.395, 95% CI −0.639 to −0.152; *P* = 0.002), and larger household size was associated with lower SE (B = −0.155 per additional member; *P* < 0.001). Family support (B = 0.150, 95% CI 0.106–0.193; *P* < 0.001) and regular exercise (B = 0.770, 95% CI 0.520–1.020; *P* < 0.001) were significantly and positively associated with SE. Compared with very high HDI countries, participants from medium, high and low HDI settings reported higher SE (B = 0.658, 95% CI 0.223–1.092; *P* = 0.003).

To confirm that estimates were not dependent on the domain-based selection procedure, a fully adjusted model entering all a priori variables simultaneously (forced entry; *N* = 964) was fitted. This model explained a comparable proportion of variance (R² = 0.225) and reproduced the primary findings, with the same factors retaining significance at near-identical effect sizes and acceptable multicollinearity (all VIF < 5; maximum VIF 4.1; ∼44 observations per factor). Differences were minor and confined to this specification (HDI category attenuated to a non-significant trend, *P* = 0.063; OM reached significance, B = −0.513, *P* = 0.037), and did not alter the substantive conclusions (Supplementary Table S4B).

### Self-efficacy and patient-reported outcomes

The PRO model explained 48.3% of SE variance (R^2^ = 0.483, *P* < 0.001) (Supplementary Table S4C). Resilience was most strongly and positively associated with SE, with each unit increase in BRS associated with a 0.43-point increase in SE (B = 0.431, 95% CI 0.306–0.556; *P* < 0.001). Better physical function (PROMIS PF4a; B = 0.094; *P* < 0.001) and mental health (PROMIS GMH; B = 0.061; *P* < 0.001) were also significantly and positively associated with SE. Pain and fatigue showed comparable negative associations, with SE decreasing by ∼0.14 points per unit increase on their respective VAS (Fatigue, B = −0.143; Pain, B = −0.145; both *P* < 0.001).

### Cluster analysis of self-efficacy

Multivariate clustering of SEMCD scores together with six PROs identified four integrated SE health profiles (Table 3, Fig. 1C). Beyond the expected low-confidence/high-burden (P1, *n* = 268, 26%) and high-confidence/low-burden (P4, *n* = 207, 20%) groups, two discordant profiles emerged. The resilient-despite-burden profile (P3, *n* = 251, 24%) was characterized by substantial physical symptom burden (high fatigue and pain, reduced physical function) yet preserved SE, above-average mental health and high resilience. Its mirror image, the low-burden/psychologically strained profile (P2, *n* = 302, 29%), showed low symptom burden but markedly reduced mental health and resilience. The four profiles differed significantly across all external validation outcomes, including life satisfaction, loneliness, mental health comorbidity, income adequacy, disease activity and IIM subtype (all *P* < 0.001), and mapped poorly onto SEMCD tertiles (adjusted Rand index 0.20), suggesting that they capture clinically meaningful multidimensional structure beyond the SE score itself (average silhouette width 0.19; elbow and silhouette criteria corroborated by Ward hierarchical clustering; Supplementary Figs S2–S4).

**Table 3:** Characteristics across the four integrated SE health profiles.

Variable	P1: low-SE/high-burden	P2: low-burden/strained	P3: resilient-despite-burden	P4: high-SE/thriving	*P*
*N* (%)	268 (26)	302 (29)	251 (24)	207 (20)	
Self-efficacy (SEMCD)	3.5 (2.3–4.5)	5.3 (4.3–6.2)	6.0 (4.8–6.8)	8.0 (7.0–9.0)	<0.001
Physical health (PROMIS GPH)	29.6 (26.7–32.4)	39.8 (37.4–42.3)	34.9 (32.4–37.4)	47.7 (42.3–50.8)	<0.001
Mental health (PROMIS GMH)	33.8 (28.4–36.3)	38.8 (36.3–43.5)	43.5 (41.1–45.8)	50.8 (45.8–53.3)	<0.001
Physical function (PROMIS PF4a)	31.9 (26.6–34.4)	36.7 (34.4–41.9)	34.4 (31.9–37.9)	45.5 (40.5–57.0)	<0.001
Resilience (BRS)	2.7 (2.2–3.2)	3.0 (2.7–3.7)	4.0 (3.3–4.2)	4.0 (3.5–4.3)	<0.001
Fatigue VAS	8.0 (7.0–9.0)	5.0 (4.0–6.0)	7.0 (6.0–8.0)	4.0 (2.0–5.0)	<0.001
Pain VAS	6.0 (5.0–8.0)	3.0 (1.0–5.0)	6.0 (5.0–7.0)	2.0 (1.0–3.0)	<0.001
Life satisfaction (SWLS)	13.0 (9.0–18.0)	17.0 (13.0–21.0)	19.0 (15.0–24.0)	26.0 (21.0–29.0)	<0.001
Loneliness (UCLA)	7.0 (5.8–9.0)	6.0 (4.0–6.0)	4.0 (3.0–6.0)	3.0 (3.0–4.0)	<0.001
Age (years)	59.0 (48.0–67.2)	55.0 (45.0–69.0)	63.0 (52.0–72.0)	60.0 (46.0–72.0)	<0.001
Female sex, *n* (%)	205 (76)	196 (65)	182 (73)	164 (79)	0.012
Active disease, *n* (%)	146 (54)	136 (45)	131 (52)	75 (36)	<0.001
Mental health comorbidity, *n* (%)	151 (56)	75 (25)	71 (28)	48 (23)	<0.001
Current income inadequacy, *n* (%)	93 (35)	29 (10)	51 (20)	4 (2)	<0.001
Most frequent subtype (%)	DM (30)	ASyS (26)	IBM (40)	DM (35)	<0.001

Data are presented as median (Q1–Q3) for continuous variables, and as *n* (%) for categorical variables. *P*-values were assessed by χ² test for categorical variables and by the Kruskal–Wallis test for continuous variables. *P* < 0.05 statistically significant. BRS: Brief Resilience Scale; IIM: idiopathic inflammatory myopathies; PROMIS: Patient-Reported Outcomes Measurement Information System; SEMCD: Self-Efficacy for Managing Chronic Disease; SWLS: Satisfaction With Life Score; VAS: visual analogue scale.

## Discussion

SE has received little attention in IIMs despite their disease burden [3, 16]. While SE has been examined in other rheumatic conditions including RA and SSc [12, 23, 24], to our knowledge this is the first large-scale study to characterize SE-associated factors and integrated SE health profiles in IIMs.

SE varied across SEMCD domains, with lower confidence in managing fatigue and physical discomfort than emotional regulation or healthcare utilization. A similar pattern has been reported in SSc, suggesting that symptom-specific confidence may represent a shared vulnerability across systemic autoimmune diseases [12]. In IIMs, this may reflect treatment-refractory muscle weakness and fatigue, which are associated with disease activity, pain and psychological burden [16]. No fatigue specific self-management programme currently exists for IIMs [28].

SE was strongly associated with several PROs, particularly overall physical health, consistent with the central role of physical symptom burden in IIMs. Positive associations with resilience are consistent with findings from RA, where resilience has been linked to fatigue, SE and QoL [29], while inverse associations with fatigue and loneliness align with previous evidence demonstrating lower self-management confidence among individuals with greater fatigue and social isolation [29–31]. Collectively, these PROs explained nearly half of the variance in SE, with resilience emerging as the strongest positive correlate, suggesting that SE may be more closely associated with psychological and functional factors than disease severity alone, consistent with observations in SSc [23].

Lower SE was independently associated with financial difficulty, active disease and mental health comorbidity, consistent with previous studies in inflammatory rheumatic diseases [13, 15, 32–34]. Greater family support and regular exercise were positively associated with SE, although the relationship with exercise is likely bidirectional. Female sex became positively associated with SE only after adjustment, suggesting confounding in unadjusted analyses. Higher SE in lower-HDI countries may reflect selection bias among more engaged respondents rather than population-level differences.

Multivariate cluster analysis identified four distinct SE health profiles that extended beyond conventional SEMCD-based stratification. One profile (P3) combined high physical symptom burden (i.e. elevated fatigue, pain and reduced physical function) with preserved SE, high resilience and above-average mental health—a pattern potentially consistent with response-shift or resilience-related coping described in chronic inflammatory conditions, though this interpretation remains speculative given the cross-sectional design. Its inverse, a low-burden/psychologically strained profile (P2), suggests low physical burden does not necessarily correspond to high self-management confidence; here, psychological factors appeared to be more closely linked to SE than physical health. Low-confidence/high-burden (P1) and high-confidence/low-burden (P4) profiles completed the picture, with P1 disproportionately represented by patients with high mental health comorbidity and financial difficulty. The four profiles differed across external validation variables and showed poor concordance with SEMCD tertiles (adjusted Rand index 0.20), suggesting that they capture multidimensional variation beyond simple SEMCD tertiles. Whether profile-tailored interventions improve outcomes requires prospective study.

Resilience and exercise were the strongest modifiable factors associated with SE, although causality cannot be inferred. Higher resilience has been associated with lower fatigue and better QoL in inflammatory rheumatic diseases [29]. Psychological interventions and exercise, including resistance training and yoga in IIM, have been associated with improved resilience and wellbeing [35–40], aligning with EULAR recommendations supporting psychological, social and self-management interventions [41].

Socioeconomic disparities highlight the importance of accessible self-management strategies. Telehealth and digital tools may improve access to monitoring and peer support [42], although implementation is limited by digital literacy and privacy concerns [28, 43–46]. Routine SEMCD assessment, taking under two minutes, may help identify patients who could benefit from additional self-management support [47].

This study has several limitations. Relying on self-reported measures may introduce recall bias, and common method variance may have inflated associations because factors and outcomes were collected within the same survey instrument; conceptual overlap between SE, resilience, fatigue, pain and mental health may also have contributed to the variance explained by the PRO model. To minimize diagnostic misclassification, participants reporting self-diagnosis or diagnosis by a non-specialist were excluded, and self-reported diagnoses were cross-checked against medication use and disease-specific clinical features. However, residual misclassification remains possible because diagnoses could not be independently verified. Nevertheless, this is, to our knowledge, the largest international patient-reported IIM cohort examining these questions. Although clinician-verified diagnoses would have been preferable, assembling a cohort of similar size and international representation would be challenging in this rare disease. Recruitment via social media and patient support networks may have introduced selection bias, potentially overrepresenting individuals with higher digital literacy, greater health engagement or better functional status, which may also explain the higher SE observed in lower-HDI settings. The cross-sectional design precludes causal inference or assessment of temporality; reverse causation cannot be excluded, and bidirectional relationships are plausible between SE and exercise, resilience, fatigue, pain and mental health. Longitudinal studies are needed to confirm these findings.

### Conclusion

This study demonstrates that SE in IIMs is associated with modifiable socioeconomic and psychological factors, with higher SE consistently associated with better physical and mental health outcomes. Four integrated SE health profiles highlight substantial patient heterogeneity, including a clinically distinctive resilient-despite-burden subgroup that would be missed by SEMCD stratification alone. These cross-sectional findings suggest potential candidates for future self-management interventions. Future research should prioritize longitudinal studies to establish temporality and clinical trials evaluating SE-targeted interventions designed specifically for individuals with IIMs.

## Supplementary Material

keag458_Supplementary_Data

## Data Availability

The data that support the findings of this study are available from the corresponding author upon reasonable request.
